# Increase on the Initial Soluble Heme Levels in Acidic Conditions Is an Important Mechanism for Spontaneous Heme Crystallization *In Vitro*

**DOI:** 10.1371/journal.pone.0012694

**Published:** 2010-09-13

**Authors:** Renata Stiebler, Anh N. Hoang, Timothy J. Egan, David W. Wright, Marcus F. Oliveira

**Affiliations:** 1 Laboratório de Bioquímica Redox, Programa de Biologia Molecular e Biotecnologia, Instituto de Bioquímica Médica, Universidade Federal do Rio de Janeiro, Rio de Janeiro, Brazil; 2 Department of Chemistry, Vanderbilt University, Nashville, Tennessee, United States of America; 3 Department of Chemistry, University of Cape Town, Rondebosch, South Africa; Wellcome Trust Mahidol University-Oxford Tropical Medicine Research Unit (MORU), Thailand

## Abstract

**Background:**

Hemozoin (Hz) is a heme crystal that represents a vital pathway for heme disposal in several blood-feeding organisms. Recent evidence demonstrated that β-hematin (βH) (the synthetic counterpart of Hz) formation occurs under physiological conditions near synthetic or biological hydrophilic-hydrophobic interfaces. This seems to require a heme dimer acting as a precursor of Hz crystals that would be formed spontaneously in the absence of the competing water molecules bound to the heme iron. Here, we aimed to investigate the role of medium polarity on spontaneous βH formation *in vitro*.

**Methodology/Principal Findings:**

We assessed the effect of water content on spontaneous βH formation by using the aprotic solvent dimethylsulfoxide (DMSO) and a series of polyethyleneglycols (PEGs). We observed that both DMSO and PEGs (3.350, 6.000, 8.000, and 22.000) increased the levels of soluble heme under acidic conditions. These compounds were able to stimulate the production of βH crystals in the absence of any biological sample. Interestingly, the effects of DMSO and PEGs on βH formation were positively correlated with their capacity to promote previous heme solubilization in acidic conditions. Curiously, a short chain polyethyleneglycol (PEG 300) caused a significant reduction in both soluble heme levels and βH formation. Finally, both heme solubilization and βH formation strongly correlated with reduced medium water activity provided by increased DMSO concentrations.

**Conclusions:**

The data presented here support the notion that reduction of the water activity is an important mechanism to support spontaneous heme crystallization, which depends on the previous increase of soluble heme levels.

## Introduction

Heme (ferriprotoporphyrin IX - Fe(III)PPIX) is an ubiquitous and essential molecule which plays key biological roles in processes like oxygen transport, respiration, photosynthesis and drug detoxification [1]–[3]. Despite this, heme is also capable of causing a number of deleterious effects in the cell [4]. Due to its amphiphilic features, heme binds to phospholipid membranes, altering their permeability and selectivity, leading to cell lysis [5]. Additionally, due to its pro-oxidant nature, heme can also generate free radicals, particularly through the decomposition of organic peroxides generating highly reactive species which mediate lipid peroxidation [6], [7], resulting in protein cross-linking [8] and nucleic acid modifications [9]. Blood-feeding organisms represent interesting models to understand adaptations developed to deal with copious amounts of heme ingested through the diet as blood. In this regard, mechanisms involved in heme degradation, binding and precipitation have been investigated [10]. In malaria parasites (*Plasmodium*) in triatomine insects [11], [12], in the helminth *Schistosoma* species [13] and others [13], heme is disposed as a dark brown crystal named hemozoin (Hz). This crystal was first described in the early 18^th^ century by Lancisi and later, Brown noted that heme was the dominant component of the so-called “malaria pigment” [14], later named Hz. It was further demonstrated that purified crystal consists only of heme molecules [15] being chemically and structurally identical to a synthetic heme crystal known as βH [16]. By means of various spectroscopic tools, it was shown that βH [17] and Hz [18] are composed of heme dimers, in which heme molecules are linked to each other by reciprocal iron-carboxylate bonds as well as hydrogen bonds between dimers [18]. The data accumulated so far points to Hz formation as a key mechanism for heme detoxification in blood-feeding organisms [11], [19], [20].

Despite the controversies over the process, recent evidence has contributed to a better understanding of heme crystallization. Noteworthy is the identification of two novel proteins involved in heme crystallization namely Heme Detoxification Protein (HDP) in the malaria parasite [21], and α-glucosidase in triatomine insects [19]. In addition, growing evidence in the literature supports the concept that lipids or amphiphilic structures (phospholipid membranes and lipid droplets) provide an environment suitable for heme crystallization [11], [12], [15], [18], [20], [22]–[24]. In this way, recent advances have identified the close association between Hz crystals and the perimicrovillar membranes (PMM), phospholipid bilayers that cover the midgut epithelium of triatomine insects [18], [20]. Also, heme crystallization supported by PMM closely follows the activity of α-glucosidase, an enzyme marker of PMM [19]. The involvement of extracellular lipid droplets, present in the gut lument of the helminth *S. mansoni* in heme crystallization has also been demonstrated [22]. In malaria parasites, Hz formation occurs physically close to lipid droplets found within the parasite digestive vacuole [23], [25]. *In vitro* studies demonstrated the effective catalytic role of organic solvents [26], lipids [24] and even artificial hydrophilic-hydrophobic interfaces [24], [20] in this process. An earlier hypothesis suggested that the thermodynamically limiting step of spontaneous heme crystallization is the solubility of heme from its acid amorphous aggregate [26], [27]. Different chemical and physical factors, such as the degree of hydrophobicity of alcohols and lipids, their ability to solubilize acid heme aggregates *in vitro*, the reduction of solution surface tension and even an increase of physical contact between heme aggregates by stirring, suggest that increased dissolution of insoluble heme aggregates is a key parameter that would modulate βH formation [24].

Here, we aimed to investigate whether increased dissolution of acid heme precipitates by reducing the medium polarity plays a role in *in vitro* βH formation. The data presented here support the notion that reduction in medium polarity increases the initial levels of soluble heme in acidic milieu, which provides a suitable environment for nucleation of βH crystals.

## Materials and Methods

### Chemicals and reagents

Hemin chloride, HEPES, sucrose and polyethylene glycol (PEG) (PEG 300; PEG 3.350; PEG 6.000; PEG 8.000; PEG 20.000; PEG 22.000) were purchased from Sigma Chemical Co. (St. Louis, MO, USA). Pyridine, acetonitrile, dimethyl sulfoxide (DMSO), sodium acetate, sodium bicarbonate, glacial acetic acid, and others reagents were obtained from (Merck, Darmstadt, Germany) and used without further purification. All other reagents were of analytical grade. All water used in the study was of ultrapure grade.

### Heme solubilization

The effect of different organic solvents on heme solubility was assessed by two different approaches: the first one by measuring the light absorption of the Soret band and the second one by using the alkaline pyridine method [28]. For both methods, 100 nmoles of heme were added to 1.0 mL of sodium acetate buffer containing DMSO, polyethyleneglycols in polypropylene (1.2 mL) tubes were shaken for 10 minutes at room temperature. Then, the tubes were centrifuged for 10 minutes at 17.500×g and the supernatants collected. For the first procedure, all the supernatants were analysed by light absorption wavelength scan between 300 nm and 800 nm, which were carried out in a GBC-920 spectrophotometer (GBC, Australia). To quantify heme, an aliquot of 300 µL from supernatants were added to 700 µL of alkaline pyridine solution (20% (v/v) of 1 M NaOH; 48% (v/v) pyridine; 32% (v/v) MilliQ water). The samples were analyzed by light absorption wavelength scan between 500 nm and 600 nm in a GBC-920 spectrophotometer (GBC, Australia).

### Heme crystallization

Spontaneous heme crystallization reactions were carried out in polypropylene tubes in the presence of 0.5 M sodium acetate buffer, pH 4.8, 100 µM hemin, previously prepared in 0.1 M NaOH as 10 mM stock solutions, in a final volume of 1.0 mL. Tubes were kept for different times at 28°C. To evaluate the role of organic solvents on heme crystallization, different concentrations of DMSO or PEGs were added previously to sodium acetate buffer before reactions were started. Then, the pH of all solutions were measured and adjusted to give an apparent pH of 4.8. The βH produced was determined by washing the pellet with “*extraction buffer*” (0.1 M sodium bicarbonate and SDS 2.5%, pH 9.1), solubilizing it in 0.1 M NaOH and measuring the amount of heme spectrophotometrically at 400 nm [11].

### Spectroscopy and electron microscopy studies

Fourier-Transform Infrared (FTIR) spectroscopy and X-ray powder diffraction (XRD) were used to confirm the identity of βH in both DMSO and PEG-driven reactions. To characterise the pigments formed by DMSO-induced reactions, FTIR analyses were carried out in dried samples homogenized in Nujol mulls. FTIR spectra were recorded between 2000 cm^−1^ and 1000 cm^−1^ in a Perkin–Elmer Paragon 1000 FT Infrared Spectrophotometer [27]. In PEG-induced reactions, the final products were purified by using 0.1M sodium bicarbonate buffer, pH 9.1 washing steps as previously described [29]. FTIR spectra of undried material were recorded between 2000 cm^−1^ and 1000 cm^−1^ and were carried out as Nujol mulls in a Thermo Mattson Satellite FTIR. XRD measurements of pigments induced by DMSO-driven reactions, were performed in undried samples using a Huber Imaging Plate Guinier Camera 670 [24] in the 2θ range 4–30° with Cu-Kα radiation (*λ* = 1.5418 Å) operating at 20 mA and 40 kV, with a step 75 resolution of 0.005°. For PEG-induced reactions, XRD analyses of purified dried pigments were performed using Cu Kα radition (λ = 1.541 Å), with data collection on a Scintage Int. (U.S.A.) instrument with vertical goniometer in the 2θ range 5–40° using a silicon sample holder. Scanning eletron microscopy (SEM) was used to investigate the external morphology of the βH produced. Finely ground samples were sprinkled onto aluminum stubs pre-coated with an almost dry carbon and glue mix. Excess sample was then removed before the samples on the stubs were sputter-coated with gold–palladium, and finally examined with a Leica S440 Scanning Electron Microscope (for DMSO-induced pigments) [27] and a Hitachi S-4200 (Japan) Scanning Electron Microscope (for PEG-induced pigments) [30].

### Osmolality measurement

Solutions with 1 and 10% of DMSO were prepared in acetic acid butter pH 4.8. Measurements were performed on a VAPRO 5520 vapor pressure osmometer (Discovery Diagnostics, Canada).

### Data analysis

Kinetics of βH reactions were analysed by using linear least-squares fitting methods with the program GraphPad ©Prism 5.0. The data were fitted according to Avrami equation [27]:
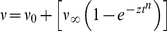
where ν is the amount of βH formed (in nmols), νο is the amount of βH present at the beginning of the reaction, ν_∞_ is the amount of βH formed at completion of the reaction, *z* is the rate constant and *n* is the Avrami constant. For a process in which growth occurs along an interface between the two interconverting phases, as is likely to be the case for βH formation in this model reaction, n takes an integer ranging between 1 and 4. Water activity analyses were done by using the following equation [31]:
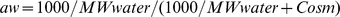
where MW*water* is the molecular weight of water and C*osm* is expressed in Osmol kg^−1^.

Comparisons between groups were done by the non-paired Student's t-test or one-way ANOVA analysis of variance and *a posteriori* Tukey's test for pairwise comparisons. The results were expressed as mean ± standard error and considered significantly different at *p*<0.05 as indicated in figure legends. Student's t-test, ANOVA, Tukey's test and correlation analysis were performed by GraphPad Prism 5.0 software.

## Results and Discussion

### DMSO promotes heme solubilization and further crystallization into βH under acidic conditions

**Figure 1:**
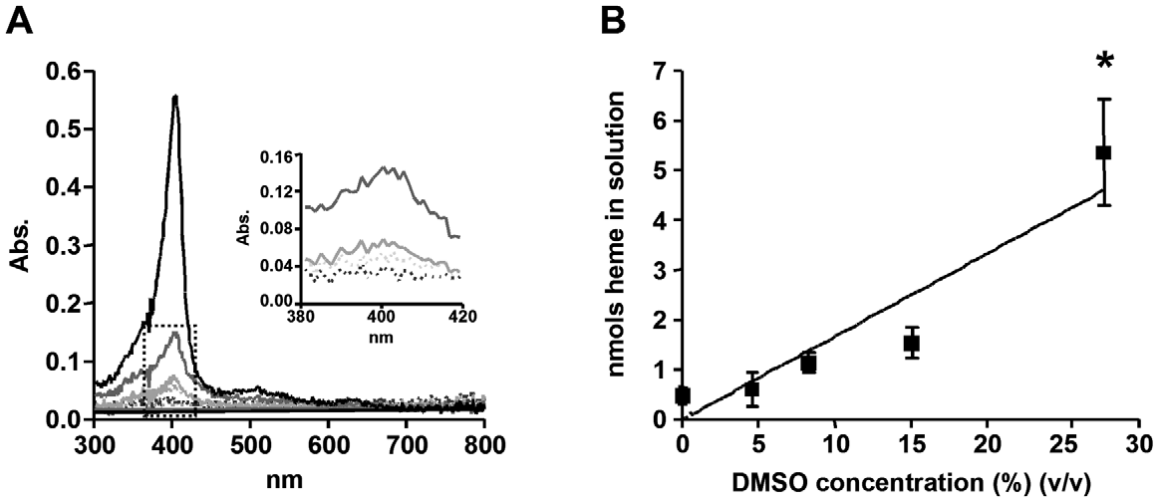
DMSO promotes spontaneous heme solubilization in acidic conditions. (A) Different concentrations of DMSO in 0,5 M sodium acetate buffer pH 4.8 and 100 µM heme with a final volume of 1.0 mL were shaken for 10 minutes and centrifuged at 10 000×g. for 10 min. The supernatants were analyzed by uv-visible spectroscopy between 300 nm and 800 nm. An expansion magnification of the dotted box is shown in the inset. Dashed line black: control; dashed line gray: 4.6% DMSO; pale gray: 8.3% DMSO; dark gray: 15.1% DMSO; black: 27.7% DMSO. (B) Heme content in solution was quantified using the alkaline pyridine method. Data are expressed as mean ± SEM, of three different experiments in B.

**Figure 2:**
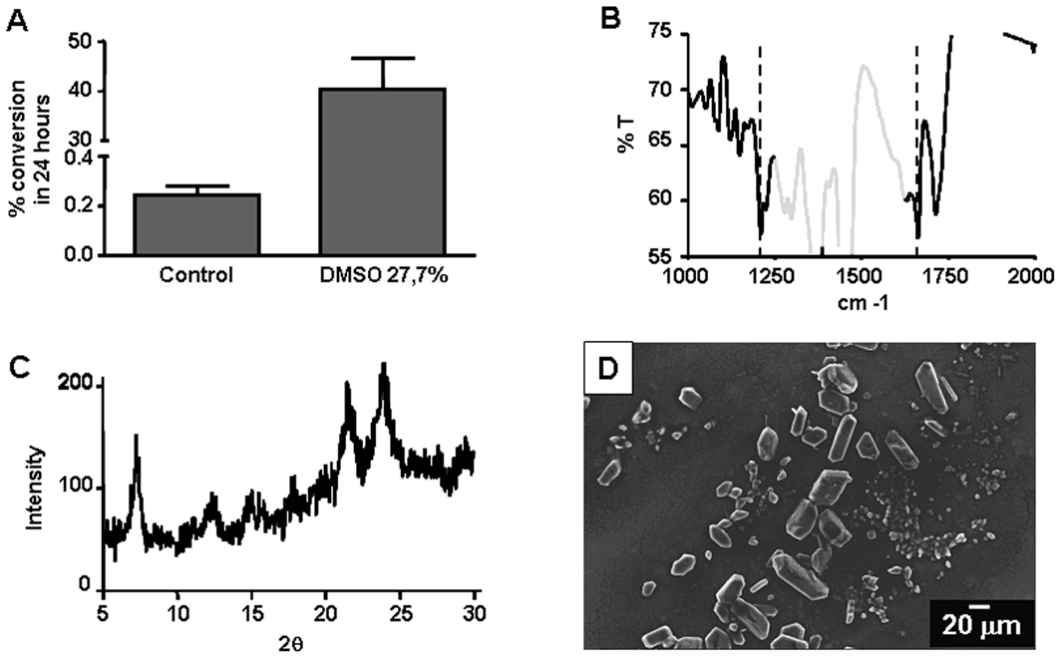
DMSO promotes spontaneous heme crystallization in acidic conditions. (A) Spontaneous heme crystallization was performed from a 100 µM solution at 27.7% v/v DMSO in 0.5 M sodium acetate buffer pH 4.8, over 24 h at 28°C. Data are expressed as mean ± SEM, of three different experiments. (B) The final reaction products were then characterized by FTIR spectroscopy. The large Nujol peaks in the region between 1320 cm^−1^ and 1550 cm^−1^ are depicted in light gray whereas the key βH peaks are shown at 1664 cm^−1^ and 1210 cm^−1^. (C) X-ray powder diffraction (XRD) was also used to confirm the identity of βH. (D) Scanning electron microscopy (SEM) was used to investigate the external morphology of the βH produced.

It is well known that, *in vitro*, heme forms insoluble amorphous aggregates under acidic conditions, which are distinct from βH crystals [24]. However, inside the *Plasmodium* food vacuoles, Hz represents, by far, the dominant form of heme, comprising at least 95% of all iron content in that compartment [32]. This indicates that, upon relase from hemoglobin, heme does not form amorphous aggregates within the food vacuole, being rapidly and efficiently converted into Hz crystals [33]. Among the physico-chemical requirements for heme crystallization, a pH close to the heme pK_a_ (4.8) is of foremost importance [16], [20], [27] and is close to the physiological pH of blood digestion in Hz-producing organisms [34], [35]. However, an interesting aspect related to heme crystallization reactions conducted in aqueous medium, is that amorphous heme precipitates are slowly converted into organized βH crystals, which presumably occurs by increasing heme solubilization [26], [27], [36]. In fact, Egan and co-workers recently demonstrated that heme and water spontaneously form a complex in aqueous medium, which depends on the interaction of the carboxyl group with the central iron of heme [37]. Therefore, a proposed model suggested that, to produce βH crystals, heme dimers are formed by means of reciprocal iron-carboxylate linkage between heme molecules, which would require the displacement of the axial water molecule bound to the porphyrin [24], [27]. Strengthening this proposal, alcohols accelerated spontaneous βH formation *in vitro* [26], [38] in reactions that depend on the hydrophobicity of these compounds and their ability to solubilize heme [26]. However, spontaneous heme crystallization was also promoted by benzoic acid in a mechanism that does not involve solubilization of acid heme precipitates [37]. Therefore, to assess whether reduction of the medium polarity would influence spontaneous heme crystallization, our first step was to investigate the effect of the aprotic solvent DMSO on heme solubility under acid conditions. For this purpose, heme was incubated in the presence of various DMSO concentrations (4.6% to 27.7%) in sodium acetate buffer at pH of 4.8 over 10 minutes and then centrifuged to separate soluble heme from amorphous aggregates. Figure 1A shows the UV-visible absorption spectra of the supernatant obtained after centrifugation of heme in the presence of different DMSO concentrations, acquired between 300 nm and 800 nm. It can clearly be observed that DMSO increases the Soret absorption band of heme (around 400 nm) in a concentration-dependent manner, suggesting an increase of heme solubility in conditions of reduced medium polarity. In order to confirm this finding, quantification of heme in the supernatants by a more reliable method (the alkaline pyridine method), indicated that DMSO increased the levels of soluble heme about ten times in acidic medium (Figure 1B). Also, a positive correlation (r^2^ = 0.6480, *p*<0.0001) between the amount of solubilized heme and the DMSO concentration was achieved, strongly indicating that reduced polarity promotes heme solubilization under acidic conditions. We next investigated whether DMSO support spontaneous heme crystallization. Figure 2A shows that incubation of heme for 24 h in the presence of 27.7% DMSO strongly promoted (*p*<0.0001) the formation of an insoluble heme pigment *in vitro*. FTIR spectroscopic analyses of this material exhibited prominent transmission peaks at 1211 cm^−1^ and 1664 cm^−1^, which designate the characteristic peaks of the iron-carboxylate bonds of βH (Figure 2B). Additionally, XRD analyses of this material (Figure 2C) demonstrated the presence of sharp Bragg diffraction peaks corresponding to a crystalline material, which is structurally and chemically identical to βH [18], [39]. The morphologies of the dried products were examined by scanning electron microscopy (SEM). Previous data from literature have shown that βH and Hz crystals are very regular in shape, presenting well-defined crystal faces [11], [40]. In figure 2D, we show the external morphology of very regular crystals, which markedly resemble those of Hz [18], [27]. Curiously, the crystals produced by DMSO are extremely large compared to those found biologically [18], [27] and reached up to 55 µm in length (Figure 2D). These results unambiguously identify the product of heme preciptation induced by DMSO as true βH.

**Figure 3:**
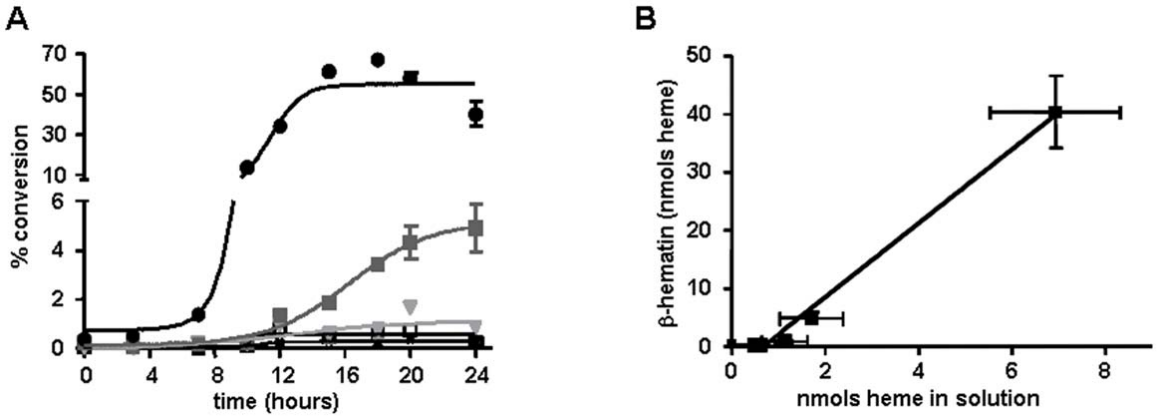
Increase in DMSO content promotes spontaneous heme solubilization and crystallization in acidic conditions. (A) Kinetics of spontaneous heme crystallization were performed at different concentrations of DMSO with heme at 100 µM over 24 h using 0.5 M sodium acetate buffer, pH 4.8 at 28°C with a final volume of 1.0 mL. Data are expressed as mean ± SEM, of least three different experiments. Open square: control; open triangle: 4.6% DMSO; inverted gray triangle: 8.3% DMSO; dark gray square: 15.1% DMSO; black circle 27.7% DMSO. (B) Correlation of spontaneous βH formation at 24 h with heme solubilization at different DMSO concentrations (r^2^ = 0.8791).

The kinetics of spontaneous βH formation was carried out in the presence of various DMSO concentrations. Figure 3A shows that βH formation exhibits a sigmoidal shaped curve and that higher DMSO concentrations facilitated heme crystallization. It can also be observed that there are no appreciable products in the control or at lower DMSO concentrations (4.6% or 8.3%). In addition, about 50% of heme was converted into βH at 27.7% DMSO 15 h after incubation, while control samples at this time point converted only 0.2%.

The Avrami equation is commonly used to model many different solid-state processes that involve nucleation and growth [27]. In this equation, (refer to methods section), the *n* values represent a function of the dimensionality of the growth process (assumed to be spherical, circular, or linear) and the type of nucleation (instantaneous or sporadic) [27]. The exponent is influenced by the type of nucleation, crystal morphology and the occurrence of secondary crystallization. Kinetics data from reactions with DMSO were fitted to the Avrami equation, and give the r^2^ values for the four possible values of the Avrami constant (*n* = 1, 2, 3 or 4), which indicate that heme crystallization performed in these conditions involves sporadic nucleation and spherical growth. For the reactions with 15.1% DMSO, a reliable fit to kinetic equations was not possible. Taking the reaction with 27.7% DMSO as an example, the best fit to the Avrami equation suggests a *n* value of 4. This indicates that DMSO promotes βH formation by sporadic nucleation (nucleation occurs throughout the process) and spherical (or 3-dimensional) growth from these nucleation points. This pattern is consistent with reactions induced by acetate and benzoic acid, as previously reported [27], [37]. The rate constant (z) for these conditions is 3.2±0.3×10^−5^ h^−4^ (or 9.3±0.9×10^−14^ min^−4^), proceeding in a much slower way than with 4.5 M acetate, where z = 2.3×10^−10^ min^−4^ at 37 °C [27] and even 0.050 M benzoic acid (4.8×10^−12^ min^−4^ at the same temperature) [37]. Therefore, these data strongly suggest that lowering the dielectric constant of medium accelerates heme crystallization *in vitro* relative to control. Just as a comparison, the rate constants obtained using total lipids extracted from regurgitates of adult *S. mansoni* females gave rate constants of 74±18 h^−2^, but Avrami constant, *n* = 2 [22]. Thus, biological heme crystallization proceeds orders of magnitude faster than those spontaneously in the presence of DMSO. Conceivably, in nature, the organic component (phospholipid membranes, vesicles or lipid droplets) mediate βH formation by eliminating the axial water bound to heme, thus allowing the heme dimer formation, as previously reported [24]. In this sense, these data provide a strong experimental support to this proposal. Finally, in order to establish a relationship between the initial heme solubilization and its subsequent crystallization induced by DMSO, in Figure 3B a linear regression analysis shows that these two parameters are strongly correlated (r^2^ = 0.8791; *p*<0.0001).

### Reduction in medium polarity by polyethers drives spontaneous heme crystallization

**Figure 4:**
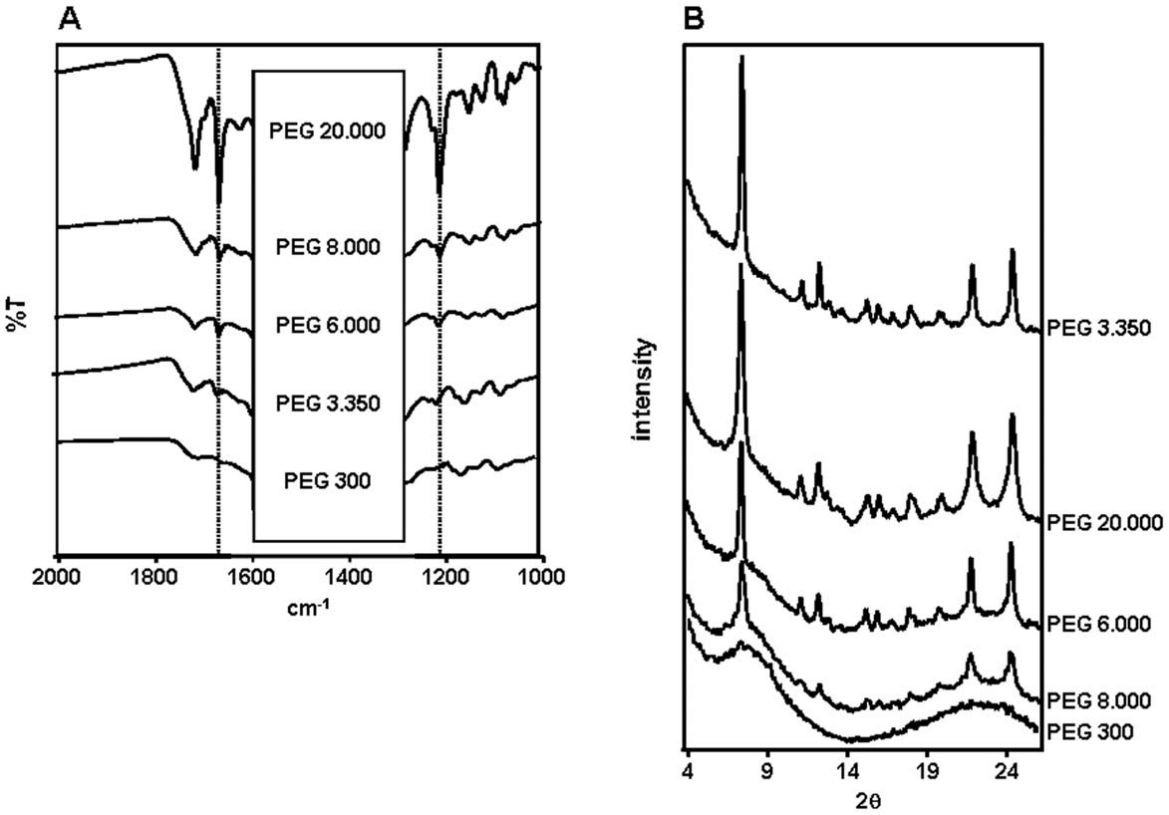
PEGs are able to induce βH formation in acid conditions. Spontaneous heme crystallization was performed in the presence of 4.7% of different PEGs at 100 µM, in 0.5 M sodium acetate buffer pH 4.8, over 5 days at 28°C with a final volume of 1.0 mL. Samples were centrifuged and the pellet washed in 0.1 M sodium bicarbonate buffer and 2.5% SDS, pH 9.1, until the solution was almost clear. (A) Pellets were then characterized by FTIR spectroscopy. The large Nujol peaks in the region between 1300 cm^−1^ and 1600 cm^−1^ are obscured by the labels, but the key βH peaks are clearly seen at 1664 cm^−1^ and 1210 cm^−1^. (B) X-ray powder diffraction (XRD) confirms the identity of βH.

**Figure 5:**
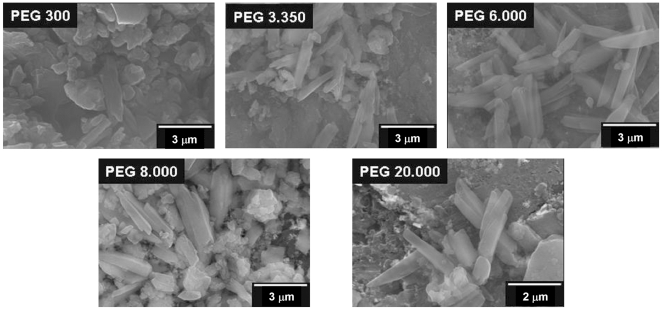
Scanning electron micrographs of βH induced by PEGs. Scanning electron microscopy (SEM) was used to investigate the external morphology of the βH crystals produced by different PEGs. Well formed crystals are seen in the presence of PEG 6.000, 8.000 and 20.000 which closely resemble hemozoin. Less regular crystals appear to be formed by PEG 3.350 and few if any are formed in the presence of PEG 300.

**Figure 6:**
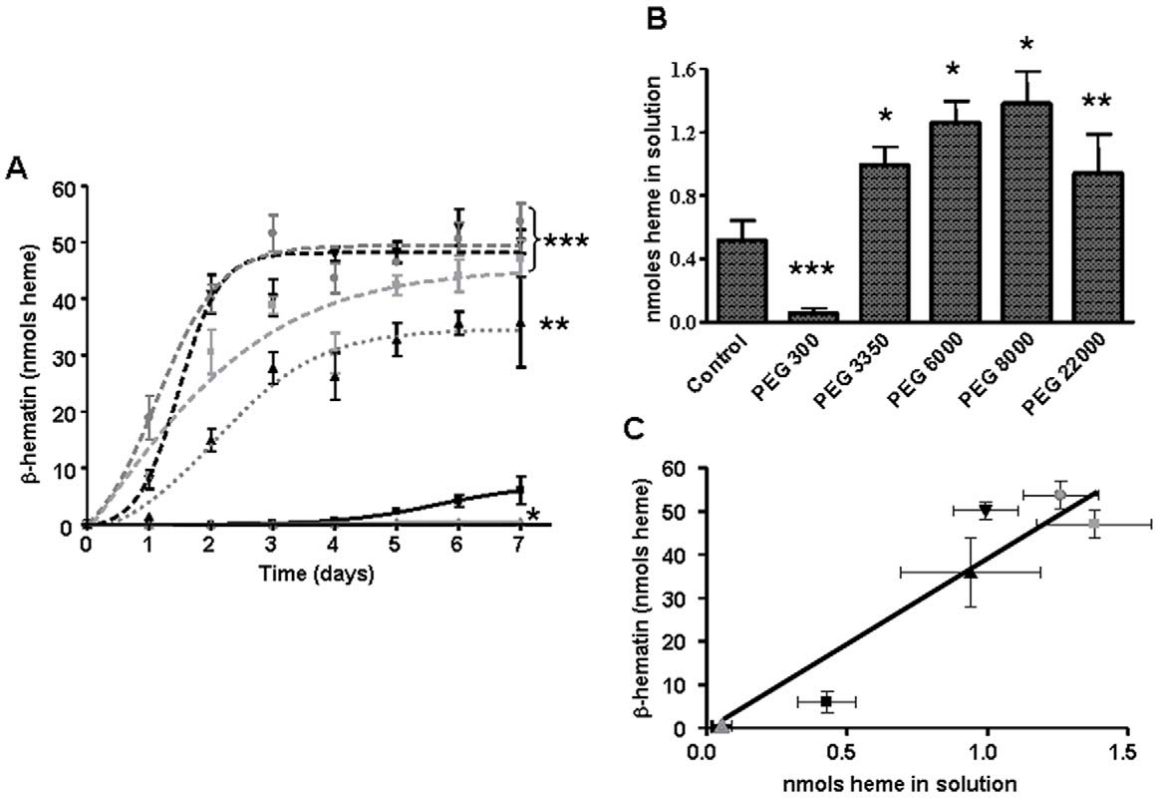
Previous heme solubilization by PEGs promotes spontaneous heme crystallization in acidic conditions. (A) Kinetics of spontaneous heme crystallization were performed in the presence of 4.7% of different PEGs with heme at 100 µM over 7 days using 0.5 M sodium acetate buffer, pH 4.8 at 28°C with a final volume of 1.0 mL. Data are expressed as mean ± SEM, of least three different experiments (^*^ p<0.05, control *vs.* PEG 300; ^**^ p<0.01, control *vs.* PEG 22.000; ^***^ p<0.0001, control *vs.* PEG 3.350, PEG 6.000, PEG 8.000). Black square: control; gray triangle: PEG 300; inverted black triangle: PEG 3.350; gray circle: PEG 6.000; gray square: PEG 8.000; black triangle: PEG 22.000. (B) Heme content in solution was quantified by the alkaline pyridine method. Data are expressed as mean ± SEM, of three different experiments in A and B, (^***^ p<0.05, control *vs.* PEG 300, ^**^ p<0.01, control *vs.* PEG 22.000 and ^*^ p<0.0001, control *vs.* PEG 3.350, PEG 6.000 and PEG 8.000). (C) Correlation of spontaneous βH formation after 7 days and heme solubilization in the presence of different PEGs (r^2^ = 0.8940). Black square: control; gray triangle: PEG 300; inverted black triangle: PEG 3.350; gray circle: PEG 6.000; gray square: PEG 8.000; black triangle: PEG 22.000.

To gain insight into the physico-chemical requirements of spontaneous βH formation, we performed heme crystallization reactions in the presence of different polyethers, such as polyethylene glycols (PEGs) of six different masses (300, 3.350, 6.000, 8.000, 20.000 and 22.000) at 4.7% (w/v) final concentration. Figure 4A shows the FTIR spectra of pigments isolated after five days of reactions induced by PEG 300, 3.350, 6.000, 8.000 and 20.000. The characteristic βH transmittance peaks at 1210 cm^−1^ and 1664 cm^−1^ were seen in all PEG-derived samples, with the exception of PEG 300. Interestingly, this pattern was also observed in corresponding XRD traces of all these pigments, in which the 7.4°, 21.7° and 24.3° Bragg diffraction peaks characteristic of βH are present in all PEG-derived pigments, but not in the PEG 300 samples (Figure 4B). Thus, similarly to DMSO, polyethers with molecular weigth higher than 300 Daltons are able to trigger heme crystallization. The morphologies of these dried pigments induced by PEGs were observed via SEM. Figure 5 shows a high proportion of brick-shaped crystalline structures in reactions performed in the presence of PEGs 3.350, 6.000, 8.000 and 20.000 and in a much lesser extent in PEG 300. Although some of the dried heme reaction products of PEG 300 resembled crystals superficially, the XRD pattern shown in Figure 4B unambiguously demonstrate that this material is amorphous. The surface appearances of these crystalline structures are quite similar, if not identical, to those exhibited by natural Hz [18] and βH [27]. Compared to the crystals produced by DMSO (Figure 2D), the crystal's size produced by PEGs was considerably smaller exhibiting the classical regular brick-shaped crystals with lengths ranging between 2.5 µm (for PEG 3.350) and 3.91 µm (for PEG 6.000). The density of regular brick-shaped crystals also varied between 5.7 crystals/field (for PEG 20.000) and 27 crystals/field (for PEG 6.000). A kinetic investigation of PEG-induced βH formation was conducted over seven days. Figure 6A shows that in the absence of any additive (control), heme crystallization becomes evident after five days of reaction. However, even after 7 days, the reaction has not proceeded far enough to allow a reliable fit from kinetic equations. Also, four, out of five PEGs investigated, were able to produce large quantities of βH. Interestingly, only polyethers with molecular weight higher than 300 Daltons were able to induce significant (*p*<0.05) heme crystallization. As demonstrated in figures 4 and 5, incubation of heme with PEG 300 did not result in substantial βH formation, and in figure 6A we could observe that in fact this polyether specifically inhibited the process, as the amount of crystals after 7 days was significantly lower (*p*<0.05) than the control. In the case of PEG 3.350 the kinetics appear to conform to *n* = 3 or 4, as is the case in acetate [27], benzoate [37] and aqueous DMSO (with the same interpretation as indicated above for DMSO). Since there is almost no difference in the fit of *n* = 4 and 3, we have opted for the former to be consistent with the other systems. The r^2^ values and the rate constants for βH formation in the presence of PEGs are shown in Table 1. It is important to notice that direct comparison of the rate constants for reactions with different *n* values is not possible. The data show that kinetics in the presence of 4.7% PEG 3.350 is considerably lower than in 27.7% DMSO (z = 3.7±0.8×10^−7^ h^−4^ *vs.* 3.2±0.3×10^−5^ h^−4^). Other PEGs that were tested promoted βH formation, showing best fits with *n* = 2, which can be interpreted in two ways, being either instantaneous nucleation (all nucleation sites present at the beginning of the process) and two-dimensional (disc-like) growth, or sporadic nucleation and linear (presumably needle-like) growth [27]. The latter would seem more likely in this case since it is difficult to imagine that PEGs would provide direct nucleation sites for epitaxial growth (which is thought to happen with lipids) [24]. In fact, the data shown in figure 5 supports this concept since the crystal's morphologies are clearly elongated. The reactions triggered by acetonitrile gives a best fit with *n* = 2, very similar to those found for three PEGs (data not shown). An important observation to consider in the kinetic studies is related to the autocatalysis of βH formation, which was proposed to be one of the mechanisms of crystal production, based on the observation that purified βH could itself promote crystal growth as described in the literature [41]. However, in all systems tested in this work (Figures 3A and 6A) and in others [20], [27], the reactions become less efficient in later times.

**Table 1:** Values of r^2^ for different values of n and rate constants for β-hematin formation in the presence of PEGs.

	r^2^				Rate constant a
	n = 1	n = 2	n = 3	n = 4	k/h^−n^
**PEG 3.350**	0.8589	0.8924	0.8984	0.8976	3.7±0.8×10^−7^
**PEG 6.000**	0.8540	0.8723	0.8576	0.8358	8±1×10^−4^
**PEG 8.000**	0.8773	0.9036	0.8972	0.8897	4.7±0.7×10^−4^
**PEG 22.000**	0.6979	0.7177	0.7102	0.7046	2.4±0.6×10^−4^

a n = 4 for PEG 3.350 and n = 2 for other PEGs.

Similar to the DMSO reactions, PEG increased heme solubilization in acidic conditions. Figure 6B show that PEG 3.350, 6.000, 8.000 and 22.000 significantly increased the levels of soluble heme compared to control. Also, PEG 300 significantly (*p*<0.05) reduced heme solubility in acidic conditions, re-inforcing the concept that initial solubilization is an important requirement to allow further heme crystallization *in vitro*. Figure 6C show a linear regression analysis indicating that, similarly to DMSO, βH formation and initial heme solubilization are strongly correlated (r^2^ = 0.8940; *p*<0.0001).

Despite the differences on the *n* values of the Avrami equations, it is possible that chemical and biological heme crystallization would not share the same mechanism. In this way, the recently described HDP in *Plasmodium* [21] and the alpha-glucosidase in *Rhodnius* [19] may represent novel catalysts of this process *in vivo*, which would not involve previous heme solubilization. However, assuming that hemoglobin digestion and heme release occurs distantly from the sites where heme is converted to Hz, especially in *Schistosoma* and in triatomine insects where both processes occur extracellularly [22], [42], an environment that allow a suitable diffusion of heme, by providing initial high levels of soluble heme in acidic conditions, would be essential for optimum nucleation of Hz formation *in vivo*. In fact, preliminary results by our group strongly indicates that the levels of soluble heme drop as βH formation induced by DMSO 27.7% proceeds, suggesting that the source of heme to nucleate crystal formation comes from the soluble heme pool, and not from the amorphous heme aggregates (data not shown). This also indicates that heme solubility in acidic conditions is a key parameter only for the nucleation process and not for the crystal extension, which occurs at later times of reaction with no apparent change in the soluble heme levels. Moreover, previous evidence from our group indicate that, in *R. prolixus* midgut, the physiological levels of “*free*” heme are very low since at least 97% of all iron-containing species present in that compartment is Hz [20]. This point deserves further attention and is currently being investigated by our group.

**Figure 7:**
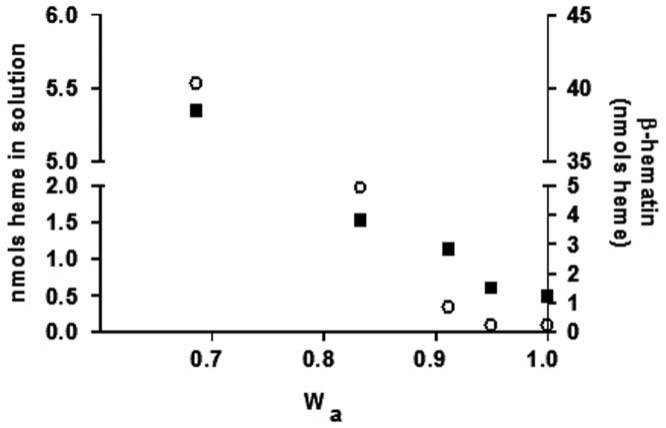
Reduction in water activity drives both heme solubility and βH formation under acidic conditions. Values of heme in solution were obtained from Figure 1B and values of βH produced was obtained from Figure 2A. Black square: nmols heme in solution; open circle: βH. Water activity was calculed based on values obtained in Dupont and Pougeois, 1983 [43].

Molecular dynamics simulations demonstrated that H_2_O–ferriprotoporphyrin IX molecules interact quite fast, producing the βH precursor by means of reciprocal iron-propionate attraction between heme molecules [24]. Then, conversion of this precursor to βH dimer would require only a ligand exchange process with bond formation from the propionate to iron and consequent displacement of H_2_O from the opposite face of each porphyrin. Since βH dimers rapidly form hydrogen bonds between the protonated propionic acid groups, it seems unlikely that such interactions are expected to occur in water because of competitive hydrogen bonding [24]. Therefore, to gain insight into the role of water in heme crystallization, we measured the medium osmolality in acetate buffers containing 1% or 10% DMSO. We observed that in the absence of DMSO, osmolality was 86.6 mmol/kg whereas in 1% and 10% DMSO the values were 213.3 mmol/kg and 1448.6 mmol/kg, respectively. These values were used to estimate the water activity in these solutions as previously reported [31]. In fact, the estimated water activity in 1% DMSO was 0.9961 and 10% DMSO was 0.9745 (data not shown). Finally, using reported water activity values of aqueous DMSO solutions at pH 7.4 [43], we could compare the degree of heme solubility and the amount of βH produced. Figure 7 shows that decrease in water activity is positively correlated with heme solubility (r^2^ = 0.9001, *p*<0.05) and βH formation (r^2^ = 0.8317, *p*<0.05).

Recently, Huy and colleagues [26] showed that induction of βH formation by alcohols is related with their degree of hydrophobicity and to their ability to solubilize heme, suggesting that dissolution of aggregated heme, and consequently the increase of heme monomers, are key physico-chemical factors in βH formation. In addition, alcohols can reduce the surface tension of a solution, thus lowering the energy barrier for creating critical nuclei [26]. Dorn and colleagues also observed that in acetate concentrations lower than 4.5 M, spontaneous βH formation was too slow to account for biological heme crystallization [44]. This is probably because acetate solubilizes hematin in acidic solution, and there is an indication that both the nucleation and linear growth rates of βH depend on the acetate concentration [27]. However, despite the fact that acetate increases heme solubility, this would not reach the levels required to drive substantial βH nucleation since our kinetic studies demonstrate spontaneous crystallization only after five days of reaction (Figures 3A and 6A). Only compounds that reduce the water content in a given concentration in the medium exhibited clear inducible effects on βH formation *in vitro* (Figures 3B and 6C). Conceivably, once hemoglobin is digested by proteases, and heme molecules released, these must reach a critical concentration in solution to allow its further crystallization that is provided by amphiphilic structures such as lipid droplets in *S. mansoni* [22] and in *Plasmodium* [23], [45] or by phospholipid membranes in *R. prolixus* [11], [12], [20]. These biological and chemical hydrophilic-hydrophobic interfaces would act by allowing heme accumulation at the surface of these structures, which would then favour the contact between heme molecules in an environment chemically suitable to drive the nucleation of unit cells of βH [46].

In conclusion, the present study demonstrates that reduced medium polarity increases heme solubility under acidic conditions, which drives the nucleation of βH crystals *in vitro*. These data represent a significant advance for understanding the mechanisms involved in heme crystallization and may open new perspectives for the rational intervention of this process.
